# Acute pancreatitis due to afferent loop obstruction treated by endoscopic ultrasound-guided transpancreatic afferent limb drainage

**DOI:** 10.1055/a-2802-4279

**Published:** 2026-03-16

**Authors:** Takashi Ito, Tsukasa Ikeura, Ayaka Takaori, Koh Nakamaru, Masataka Masuda, Shinji Nakayama, Makoto Naganuma

**Affiliations:** 1 12880 Third Department of Internal Medicine, Division of Gastroenterology and Hepatology, Kansai Medical University, Osaka, Japan

Afferent loop obstruction (ALO) is a rare mechanical complication occurring in 0.3–1.0% of patients after gastrectomy 1 . Elevated intraluminal pressure in the afferent loop can lead to cholangitis or pancreatitis. Recently, endoscopic transluminal stent placement using balloon-assisted endoscopy 2 or endoscopic ultrasound (EUS 3 ) has been reported as an effective treatment. We describe the first successful case of the endoscopic treatment of ALO complicated with acute pancreatitis by an EUS-guided transpancreatic approach ( Video 1 ).

Endoscopic ultrasound-guided transpancreatic afferent limb drainage.Video 1

**Fig. 1:**
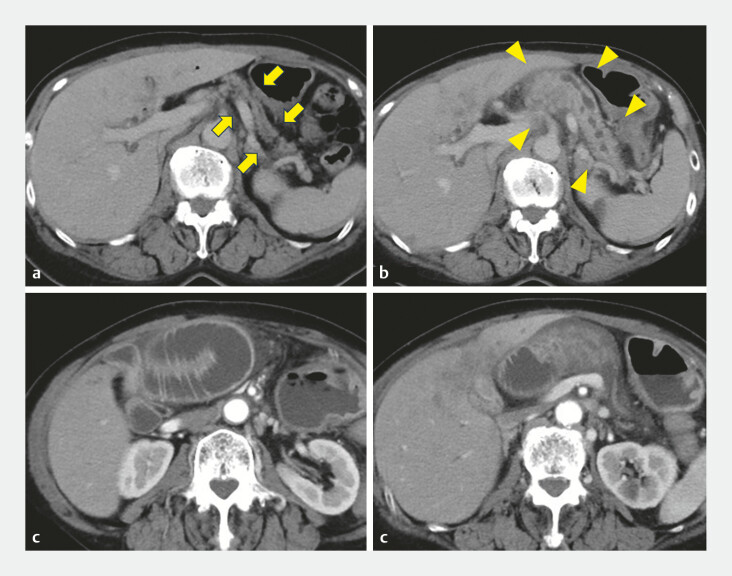
Computed tomography performed 3 months before admission and at the time of hospitalization: **a** pancreatic parenchymal atrophy 3 months before admission (yellow arrow); **b** swelling of the pancreatic parenchyma and dilated main pancreatic duct (yellow arrowhead); **c, d** dilation of the afferent loop at the blind end.

A 71-year-old woman with a history of gallbladder cancer who had undergone pancreaticoduodenectomy 5 years earlier presented with fever and abdominal pain. Computed tomography revealed dilation of the afferent limb and pancreatic enlargement with dilation of the main pancreatic duct (MPD) to 6 mm, compared with images obtained 3 months earlier ( Fig. 1 ). She was diagnosed with acute pancreatitis secondary to ALO.

**Fig. 2:**
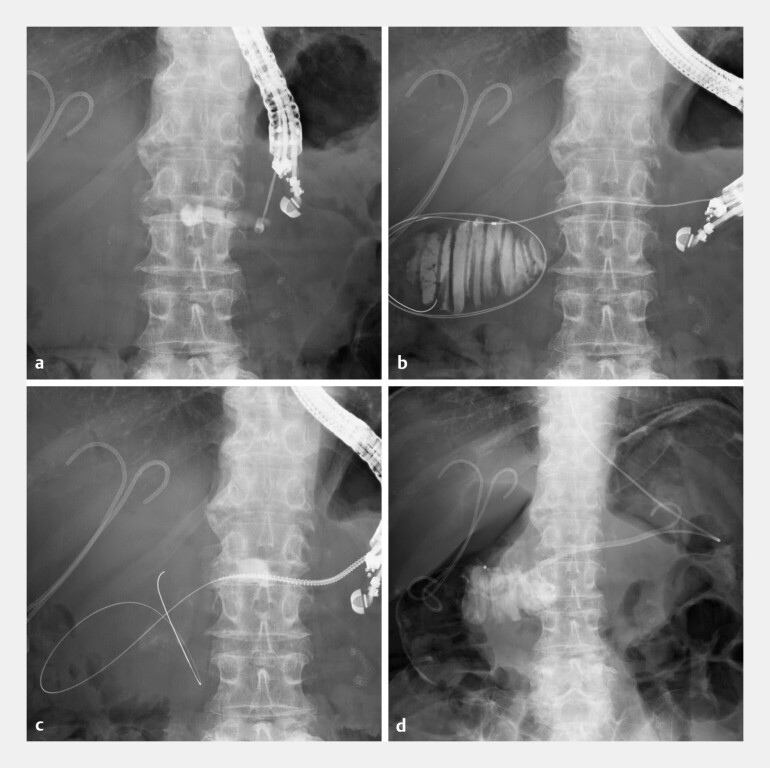
**a–d** Placement of a plastic stent and a nasodrainage tube into the dilated afferent loop at the blind end via the main pancreatic duct using endoscopic ultrasound-guided transpancreatic afferent limb drainage. **a** Under EUS guidance (UCT260; Olympus Co., Ltd, Tokyo, Japan), a 19-gauge needle (SonoTip Pro Control; Medico’s Hirata Inc., Osaka, Japan) was inserted into the dilated main pancreatic duct. **b** A 0.025-inch guidewire (Visiglide2; Olympus) and an MTW catheter (ABIS, Tokyo, Japan) were advanced across the pancreaticojejunal anastomosis into the blind end. **c** The fistula was dilated using a drill dilator (Tornus ES; Asahi Intecc, Aichi, Japan). **d** A plastic stent (6-Fr 7-cm, pigtail type, Zimmon; COOK Medical, Bloomington, IN, USA) and a nasodrainage tube (5Fr; Create Medic) were placed into the dilated blind end. EUS, endoscopic ultrasound.

**Fig. 3:**
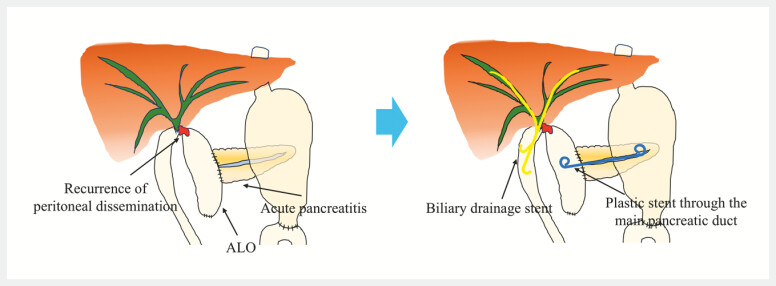
Schematic illustration of EUS-guided afferent limb drainage via the main pancreatic duct. EUS, endoscopic ultrasound.

A short-type double ballon endoscope (EI-580BT; Fujifilm, Tokyo, Japan) reached the choledochojejunal anastomosis; however, the dilated blind end where the pancreaticojejunal anastomosis was located could not be accessed due to peritoneal dissemination. Histopathological examination of endoscopic biopsy specimens revealed adenocarcinoma. As a result, decompression of the afferent limb was not achieved, and only biliary stents were placed. Consequently, we planned to perform EUS-guided transpancreatic afferent limb drainage. Under EUS guidance, a 19-gauge needle was inserted into the MPD, and a guidewire was advanced across the pancreaticojejunal anastomosis into the blind end. There was no pancreatic duct stricture. The fistula was dilated using a drill dilator. A plastic stent and a nasodrainage tube were placed into the dilated blind end ( Fig. 2 ). Six days after the procedure, the nasodrainage tube was removed because both afferent loop dilation and pancreatitis improved promptly ( Fig. 3 ). No recurrence of pancreatitis was observed without removing the stent until the patientʼs death.

EUS-guided transpancreatic afferent limb drainage may represent a feasible and effective endoscopic treatment for ALO associated with acute pancreatitis.

Endoscopy_UCTN_Code_CCL_1AC_2AH Endoscopy_UCTN_Code_TTT_1AS_2AK Endoscopy_UCTN_Code_TTT_1AS_2AI
